# The Application of Cocaine to the Eye as an Anæsthetic

**Published:** 1885-02

**Authors:** Karl Koller

**Affiliations:** House Physician to the Allgemeines Krankenhaus of Vienna


THE CHICAGO

MEDICAL JOURNAL AND EXAMINER.

VOL. L. FEBRUARY, 1885. No. 2.

### TRANSLATIONS.

THE APPLICATION OF COCAINE TO THE EYE AS AN ANÆSTHETIC. *By* DR. KARL KOLLER, *House Physician to the Allgemeines Krankenhaus of Vienna.** *Translated for the* CHICAGO MEDICAL JOURNAL AND EXAMINER *by* BOERNE BETTMAN, M. D., *Chicago.*

The object of this paper will be to report the results of some experiments relating to the anæsthesia of the eye. This is certainly not the first communication I have furnished on this subject; I sent an essay to the Convention of German Oculists, which met in Heidelberg, September 15–16, 1884, in order to claim priority.

Dr. Brettauer, of Trieste, had the kindness to make known my communications to the convention and demonstrated my experiments, which have been repeated and verified quite often in different places in Germany.

That cocaine, the alkaloid contained in the leaves of the Erythroxylon Coca, possesses the characteristic of anæsthetiz-

*Paper read in the K. K. Society of Physicians, October 17, 1884.

ing the mucous membrane of the tongue upon local application, is a fact discovered in 1859, by Nieman, the scholar of Wöhler. Its powers were made known to us in 1862 by Professor Schroff, who made mention of it for the first time in a paper read before this very Society.

It is furthermore known that cocaine diminishes the caliber of the peripheric blood vessels, by way of the circulatory system, as is also known that cocaine produces dilatation of the pupil by the just alluded to manner as well as by local application. The instillation of cocaine into the eye has therefore been demonstrated before, but those symptons which will form the main object of to-day's paper have thus far escaped attention.

Experiments made with repeated internal applications of cocaine have been invariably so unsuccessful that the remedy fell into discredit and was eventually forgotten. In 1880, Dr. von Alrep * published an extensive work on his experiments with cocaine, at the end of which he hints at the possibility of cocaine becoming a useful local anæsthetic. My colleague, Dr. Sigmund Freud,† of the "Allgemeines Krankenhaus," of Vienna, brought cocaine into prominence by his thorough compilation and interesting therapeutic work.

I started out with the supposition that a substance which paralyzes the terminations of the sensitive nerves of the mucous membrane of the tongue, would produce the same effect upon those of the cornea and conjunctiva and made a series of experiments in Professor Stricker's laboratory, based on these conjectures. My results were in short as follows:

If a few drops of an aqueous solution of Cocainum Muriat-

---

*Pflüger's Archiv. f. d. ges. Phys. 21. Bd.

† Centralbl. f. Therapie V. Heitler. August heft, 1884.

icum ‡ be applied to the cornea of a guinea pig, squirrel or dog, or by the ordinary method instilled into the *cul de sac* of the conjunctiva, the animal will wink a short time, presumably in consequence of a slight irritation, but within one-half to one minute will again open its eye, which has gradually assumed a strange staring appearance. If the cornea be now touched with the head of a pin (in order to guard against mistakes care should be taken not to come in contact with the eye-lashes), no reflex closure of the lids will ensue, the eyeball remains immobile, the head is not drawn backwards as would naturally have otherwise followed, the animal remains perfectly quiet, and the application of stronger irritants will prove conclusively that both cornea and conjunctiva are entirely anæsthetized. Thus I scratched and punctured the cornea with my needle and irritated it by means of the induced current, which was so strong that it pained the fingers and was unbearable upon application to the tongue; I cauterized the corneæ with lunar caustic until they became milky white—all this did not elicit the slightest movement from the animal.

The last two experiments convinced me that the anæsthesia affected not only the upper surface of the cornea but the entire substance. But when the cornea was cut into, at the same moment when the aqueous humor escaped and the iris prolapsed, the animal gave vent to lively manifestations of pain. I was unable to determine in my experiments on animals, whether the iris could be anæsthetized by instilling some of the solution into the corneal wound or by continued application into the conjunctival sac instituted for some time previous to the operation; for experiments relating to tests of sensibility on

‡ Cocainum Muriaticum is soluble up to 5 per cent. in water without the addition of acid, but is always opalescent. Addition of acid is to be avoided since the slightest quantity produces a severe burning sensation. The opalescent fluid is rendered perfectly clear upon filtration.

unanæsthetized animals, if only comparatively complicated, are very unsatisfactory.

I furthermore tried to ascertain whether cocaine could induce anæsthesia of an inflamed cornea. This question was decided affirmatively through experiments made on animals on whom I had previously produced a traumatic keratitis, their corneæ were as thoroughly anæsthetized as healthy ones.

The complete anæsthesia effected by a 2 per cent. solution lasts on an average ten minutes.

After these successful experiments upon animals, I no longer hesitated to test its action on the eyes of human beings, and applied it to my own eye, to that of my colleagues, and later on used it on numerous other individuals. All, without exception, testified to the complete anæsthesia of their corneæ and conjunctivæ. The course of manifestations is as follows: if a few drops of a 2 per cent. solution be instilled into the conjunctival sac or allowed to flow over the cornea a slight burning sensation and quite a copious flow of tears will ensue, which ceases in one-half to one minute, to be succeeded by a dull sensation of dryness. To an observer the eye will appear dull, staring, as noted in the animals used for experimental purposes. This expression is due to a decided expansion of the lid fissure, a phenomenon to which I will refer later on. If, now, the cornea be touched with a needle, no sensation either of pain or touch is experienced, even the reflex action fails to appear. The same applies to the conjunctiva, where also the sensation due to change of temperature is counteracted. The conjunctiva bulbi can be grasped with a mouse-toothed forceps without causing the patient the slightest discomfort, or the cornea may be indented with a probe; the only cognizance of this manipulation being indistinct vision, due to the altered curvature of the cornea. This complete anæsthesia lasts from seven to ten minutes, and gradually passes into the normal state through a long transition stage of diminished sensibility. The pupil begins to dilate about fifteen to twenty minutes after the instillation. The dilatation reaches its maximum during the first hour, diminishes visibly in the second, and a few hours later all traces of mydriasis have disappeared. The dilatation is never maximal, and the pupil reacts promptly to light and to convergence during the entire time, in consequence of which the blending sensation which always accompanies atropine mydriasis is never experienced or is present only in a very moderate degree.

The mydriasis is accompanied by a transitory and slight paralysis of accommodation; in my case and in another individual the near point receded one-half inch.

I have noticed, furthermore, a decided ischaæmia of the normal conjunctiva, especially of the conjunctiva palpebrarum, but am unable to state anything definite regarding its duration. I refrain from mentioning, for the present, certain still undetermined observations, as for instance, those referring to the ophthalmoscopic appearances, but desire to emphasize the fact that the application of cocaine during my entire observations has never been followed by symptoms of irritation.

I regard the dilatation of the lid fissure, which occurs simultaneously with the anæsthesia of the cornea and conjunctiva, due to the loss of sensibility of these parts, rendering them insensible to the irritation which in the normal state affects both cornea and conjunctiva, and which regulates the width of the lid fissure.

There are still a few practical points relating to the anæsthesia which I wish to emphasize:

1. The anæsthetic action of cocaine can be augmented; if, on the abatement of the anæsthesia, cocaine be newly instilled a second anæsthesia is induced, which lasts longer than the first. In this way I have obtained complete anæsthesia, lasting from fifteen to twenty minutes, by repeating the instillation every five minutes for a certain time.

2. The anæsthetic action is a purely local one.

3. Since it is known that cocaine is absorbed, and on every instillation a small amount enters the eye, at first into the anterior chamber, one might surmise that also the more remote parts of the eye would be anæsthetized if it were possible to introduce a large enough quantity of the remedy. But as a certain length of time is requisite for the absorption, and as the duration of the anæsthesia is limited, the numbness of the cornea will already have passed away at the time iris and ciliary body are affected, the cornea will have to be anæsthetized from anew. Both of these demands can be strictly adhered to by a series of systematized and graded applications. Thus I produced a decided diminution of the sensibility of the eye, against strong pressure, after having instilled a five per cent. solution of cocaine, repeated every five minutes for a period of half an hour.

Through the extreme liberality of Professor Dr. v. Reuss, director of the clinic of the deceased Jaeger, I have been enabled during the last two or three weeks to test the action of the remedy on inflamed eyes. Dr. v. Reuss, as well as his assistants, Drs. Bochner and Dimmer, furthered my experiments in every respect, for which I express to them my sincere thanks.

My attention was directed from the very beginning to the two therapeutical uses cocaine might be put to: first, to apply it as a narcotic in painful diseases of the eye, and, second, as an anæsthetic in operations on that organ.

I expected very good results from its action with reference to the first mentioned usage, especially in corneal and conjunctival ailments, combined with pain and photophobia. I have, in fact, employed cocaine (in a two per cent. solution) on a number of patients afflicted with conjunctivitis lymphatica, with eruptions and ulcers of the cornea, and also on a patient with fasicular keratitis. All patients treated in this manner expressed a few moments after the instillation an amelioration of their subjective condition, pain ceased and photophobia decidedly diminished. But they as unanimously complained of the return of the annoying symptom two and three hours after the application of the remedy. It is reasonable to expect, however, a complete cessation or at least a diminution in intensity of the pain and photophobia upon continued application of the solution. This mode of procedure could not, as yet, be carried out.

I obtained similar results when used on a man with painful erosions of the limbus.

In like manner I would promise myself happy results from its power to influence pain in iritis, having proven, as I think, that the anæsthetic action of cocaine extends in a certain degree to the iris and corpus ciliare.

The mydriatic action, owing to its insignificance, would not come into play here; still, we might expect the cocaine to influence the pathological process by virtue of its power to diminish the caliber of the blood vessels. Perhaps its combination with atropine treatment might prove of avail. Unfortunately the opportunity has not yet presented itself to test its efficacy in this disease.

The pain, also, consequent to the cauterization of the lids with nitrate of silver, may, by previous instillation of cocaine, be entirely controlled or at least very much influenced. The majority of patients subjected to this experiment stated that they felt no pain; in a few, pain returned after a short period, but was again immediately controlled on renewed employment of the solution. A lady patient remarked that the pain was less intense than usual, but lasted longer.

My experience regarding the use of cocaine in connection with sulphate of copper treatment is limited and to a certain extent contradictory; at any rate it would have to be applied in this connection in a much stronger solution than in cauterization with nitrate of silver.

I will now consider the second applicability of cocaine, namely, as an anæsthetic in operations on the eye.

Cocaine can be put to excellent service in the removal of foreign bodies from and out of the cornea, which procedure is usually rendered difficult by the restlessness of the patient. In the great majority (about thirty cases) of so afflicted individuals I induced the anæsthesia as follows: I dropped onto the cornea of the patient, who was either sitting or standing and looking to the ground, two drops of a 2 per cent. solution; this I repeated 3–5 minutes later. All kept their eyes perfectly quiet during the digging out of the foreign body from the cornea, and when questioned as to their sensations, replied that they had felt absolutely nothing.

Cocaine was applied with the same good results in a case of tattooing of corneal scars and in a pterygium operation.

Good effects might be expected from the use of cocaine in the cauterization of corneal ulcers with a hot iron, also in cases of *punctio corneæ* and *discissio cataractæ.* Both of the last mentioned operations, which in fact consist in the fixation of the conjunctiva and puncturing the cornea, can be made, as my experience upon animals and human beings have taught, without provoking pain.

Dr. von Reuss did a staphyloma operation upon a boy and girl aged 7 and 8 years, not narcotized, but anæsthetized with cocaine. The children kept perfectly quiet and experienced no discomfort whatever.

Dr. von Reuss was kind enough to permit me to apply cocaine in several cases upon whom iridectomies and cataract operations were done. Upon the whole I would remark concerning the above that in all these cases no irritation followed its employment, which invites to further experimentation. The experiments were followed by diverse and more or less satisfactory results, according to the strength of the solution and the method of its application. The best, indeed almost perfect results were obtained in those cases where the following method of application had been strictly adhered to: during at least half an hour prior to the operation two drops of a 5 per cent. solution is instilled into the eye at intervals of 5 minutes. The patient is placed in a horizontal position, the upper lid is raised, and while he is looking towards his feet the solution is dropped on to the sclerotic above the cornea.

One of the numerous cases thus treated, a woman, upon whom the extraction of a cataract had been undertaken, when asked at each step of the operation as to her sensations, asserted that she was not cognizant of the corneo-scleral section; the grasping and excision of the iris was felt, but produced only slight pain. She reacted to none of the various acts of the operation, neither did a stupid woman, upon whom Dr. von Reuss was loth to operate owing to extreme sensitiveness.

The following case, owing to the peculiar circumstances, is worthy of interest.

A man with seclusio pupillæ of both eyes was iridectomized on the left eye, after cocaine instillation. The man was perfectly quiet during the operation and stated that he had felt nothing of the corneo-scleral section (made with a lance-shaped knife). He experienced tactile sensation when the iris was grasped and excised, but no pain. A week later the patient underwent a similar operation on the other non-anæsthetized eye. This time he wriggled and squeezed the eyelids so violently as to render the operation very much more difficult.

Although the great majority of patients upon whom such operations are made are torpid individuals who would anyway bear their pains patiently, the last mentioned case appears to demonstrate that in such cases also an anæsthetic may render good services.

